# Full wetting of plasmonic nanopores through two-component droplets†
†Electronic supplementary information (ESI) available: Experimental details about the fabrication and the priming strategies of the nanopores, the contact angle measurements on a flat gold surface, the SERS measurements of the nanopore, including the bonded and non-bonded Raman analytes, as well as the priming of commercial Klarite SERS substrates are shown here. See DOI: 10.1039/c5sc02338f


**DOI:** 10.1039/c5sc02338f

**Published:** 2015-08-04

**Authors:** Chang Chen, XiuMei Xu, Yi Li, Hilde Jans, Pieter Neutens, Sarp Kerman, Guy Vereecke, Frank Holsteyns, Guido Maes, Liesbet Lagae, Tim Stakenborg, Pol van Dorpe

**Affiliations:** a IMEC , Kapeldreef 75 , Leuven 3001 , Belgium . Email: chang.chen@imec.be ; Fax: +32 16281097 ; Tel: +32 16287794; b Department of Physics and Astronomy , KU Leuven , Celenstijnenlaan 200D , Leuven 3001 , Belgium; c ESAT , Katholieke Universiteit Leuven , Kasteelpark Arenberg 10 , Leuven 3001 , Belgium; d Department of Chemistry , KU Leuven , Celenstijnenlaan 200F , Leuven 3001 , Belgium

## Abstract

Benefiting from the prospect of extreme light localization, plasmonic metallic nanostructures are bringing advantages in many applications. However, for use in liquids, the hydrophobic nature of the metallic surface inhibits full wetting, which is related to contact line pinning in the nanostructures. In this work, we use a two-component droplet to overcome this problem. Due to a strong internal flow generated from the solutal Marangoni effect, these droplets can easily prime metallic nanostructures including sub-10 nm nanopores. We subsequently evaluate the local wetting performance of the plasmonic structures using surface enhanced Raman spectroscopy (SERS). Compared with other commonly used surface cleaning based wetting methods such as the oxygen plasma treatment, our two-component drop method is an efficient method in resolving the pinning of contact lines and is also non-destructive to samples. Thus the method described here primes plasmonic devices with guaranteed performances in liquid applications.

## Abstract 2

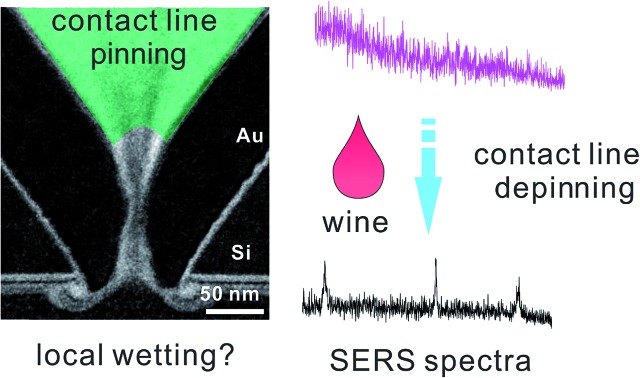
By placing a drop of wine near the sub-10 nm gold nanopore to generate a Marangoni flow, we can finally overcome the wetting problem and make the nanopore perform excellently for molecular sensing in aqueous solutions.

## Introduction

The use of surface plasmon polaritons (SPPs) excited at the interface of metallic structures and the dielectric environments is an efficient approach for focusing and manipulating photons at the nanoscale for various applications.^1^,^2^ In the past decade, many plasmonic devices have been used for local molecular sensing, especially those with small engineered gaps like bowties^3^,^4^ and nanopores.^5^,^6^ Inside these gaps, the electromagnetic field is strongly enhanced, allowing them to act as hot spots and exhibit extraordinary sensitivity for sensing. In addition, these small gaps can spatially limit the amount of analyte molecules, and may allow real-time single molecule analysis at relatively high concentrations. Different plasmonic gap structures such as the pore-cavity,^6^ the pore-bowtie,^7^ the pore-graphene,^8^,^9^ and the pore-array^10^,^11^ have been used or suggested in this context. However, in practice, ensuring full wetting of such structures is especially essential. Air bubbles trapped inside the gaps can reduce the signal sensitivity and intensity or even cause malfunctions in the plasmonic devices by preventing analyte solutions from entering the sensing zones.^12^

In general, pretreatment for full wetting is very important for using plasmonic devices in fluids. For a narrow nanochannel, when the wall of the channel is hydrophilic, it can usually be spontaneously filled by the sample liquid (*e.g.* water).^13^ The driving force for liquid imbibition in the nanochannel is the capillary pressure, and therefore improving the wettability of the channel wall will enhance the filling efficiency. The wettability of a surface is often characterized by a contact angle (*θ*), which is determined by the interfacial energy balance as described by Young's equation:

1 cos *θ* = (*γ*_sg_ – *γ*_sl_)/*γ*

where *γ* denotes the surface tension of the liquid, and *γ*_sg_ and *γ*_sl_ are the solid–gas and solid–liquid interfacial energies, respectively. As can be seen from eqn (1), a better wettability (with small *θ*) can be achieved either by increasing the surface energy of the channel wall or by using a liquid with a lower surface tension. For gold deposited nanostructures, quick accumulation of contaminants or surface hydration always results in a poor water wettability (*θ* > 80°). This hydrophobic metal–water interface becomes a critical wetting challenge.^14^,^15^ Different methods such as chemical treatments,^16^ surface modifications,^17^ and electrochemical controls,^18^ can be applied to improve the surface wettability. Universal methods based on cleaning processes like UV ozone^19^ or O_2_ plasma^20^ treatments, can usually remove the organic contaminants and temporarily render a clean gold surface with very high water wettability (*θ* < 10°). Using solvents with a low surface tension like alcohols instead of water is a common way to improve the wetting performance of devices. Additionally, eqn (1) is valid at the nanoscale too, at least for cylinder-like symmetric structures. Wetting inside a carbon nanotube was also reported.^21^

During the wetting of asymmetric structures, the morphology of the structure becomes important. A relevant example of such an asymmetric structure is a nanochannel with a sandglass-like cross-section. These kinds of asymmetric structures can be easily made by nanofabrication processes such as etching^22^ or deposition.^23^ In such nano-sandglass structures, the asymmetric part is the neck. A huge curvature at the neck may generate an energy barrier for wetting, through an effect called the contact line pinning.^24^–^26^ The contact line is the interface of liquid, gas and solid. The interface stops moving when it is pinned. Mostly, alcohols are the primary choices for wetting. During the evaporation of alcohols, there is an internal capillary flow that can move the contact line. However, this flow is still too weak to depin the contact line in asymmetric structures.

**Fig. 1:**
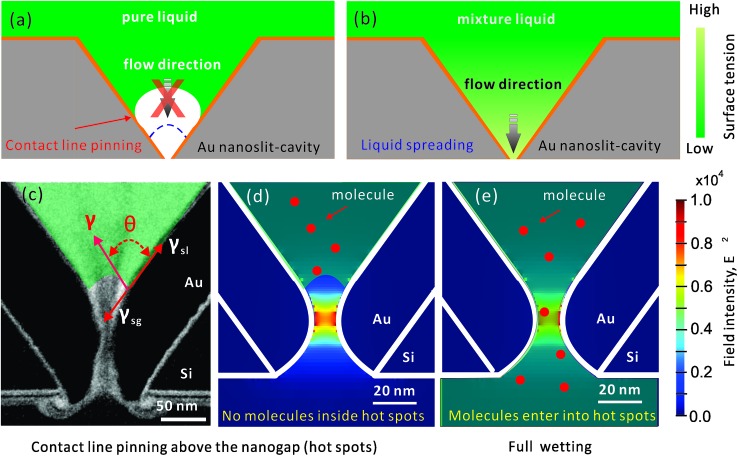
Schematic drawings of wetting inside a gold nanopore-cavity. (a) Incomplete wetting by using a pure liquid due to the contact line pinning and (b) full wetting by the Marangoni effect. The drawing is not to scale. (c) The TEM image of a 10 nm nanopore and the drawing of the distributed forces (in red) at the contact line of the solid, liquid and gas interfaces; the transparent green part represents the liquid. Hypothetical wetting status: (d) air trapped inside the nanogap prevents molecules from entering and (e) full wetting inside the nanogap opens a pathway for molecules. The numerically calculated optical field (*E*^2^) profiles are in reference to ref. 27.

In this work, we consider the use of a two-component drop to move the contact line for full wetting. A similarly shaped asymmetric nanostructure, a plasmonic nanopore (shown in Fig. 1 and S1a†) is mainly used as the platform for evaluating the wetting performance. In this structure, we have calculated in our former work that the hot spot is localized inside the sub-10 nm gap at the bottom of a 700 nm deep cavity (shown in Fig. 1d and e).^27^ Previously we have shown the high sensitivity of such nanopores for molecular sensing in air through surface enhanced Raman spectroscopy (SERS).^6^,^23^ However, it was difficult to use this kind of nanopore for sensing in an aqueous solution.^28^ Mainly this is because the nanopore is not completely wetted, and no SERS signals can be observed. On the other hand, this means we can use the intensity of SERS to evaluate the wetting status inside the nanopores. Through SERS from nanopores, we can study the feasibility of different treatments for full wetting, and perhaps to compare the efficiency of wetting strategies if the SERS can be appropriately set. Previously, to study the nanoscaled wetting behaviors inside nanopillar arrays, we have developed a method based on interferometric reflection spectroscopy.^29^ Here, to study a single nanopore channel, it is better to utilize the unique advantage of the high localization property of SERS.

## Results and discussion

### Contact line pinning inside nanopores

The incomplete wetting of asymmetric nanopores can be caused by the neck effect in the capillary rise.^26^ As shown in Fig. 1, in this nanopore structure, the surface undergoes a sharp change of the curvature at the gap, which facilitates contact line pinning above the gap. When we immerse the nanopore chip in solutions, gas bubbles will be trapped naturally in the gap (the hot spot region), which prevent the nanopore from sensing the analyte by SERS. Thus, moving the contact line across the gap is critical.

To resolve the contact line pinning problem, we introduce an efficient and non-destructive surface priming method based on the use of two-component droplets. One of the component is water, and the other is a volatile alcoholic solvent with a lower surface tension. A typical alcoholic component can be isopropyl alcohol (IPA). We chose IPA as it is safe to the objective lens and its residual adsorption on the solid surface is minimal.^30^ Different to the simple spreading of a pure liquid drop during evaporation (*e.g.*, a water drop), a two-component drop undergoes three stages: dynamic spreading, fast receding and slow receding,^31^–^35^ caused by the solutal Marangoni effect.^36^ In this mixture drop, the higher evaporation rate of alcohol can increase the surface gradient near the contact line. In particular, the formed gradient can pull the liquid from the central alcohol-rich region (with a low surface tension) to the contact line, water-rich region (with a high surface tension), and thus forms an intense liquid flow moving towards the interface, and depins the contact line (shown in Fig. 1b). This mechanism is used to explain the extraordinary spreading (of both area and speed) of an alcohol and water mixture drop on a solid surface; a well-known effect *e.g.* in the phenomenon of tears of wine.^37^ In recent work, this has been used well to control the motion of droplets mixed with two components.^38^

### Contact line moving by the Marangoni effect

**Fig. 2:**
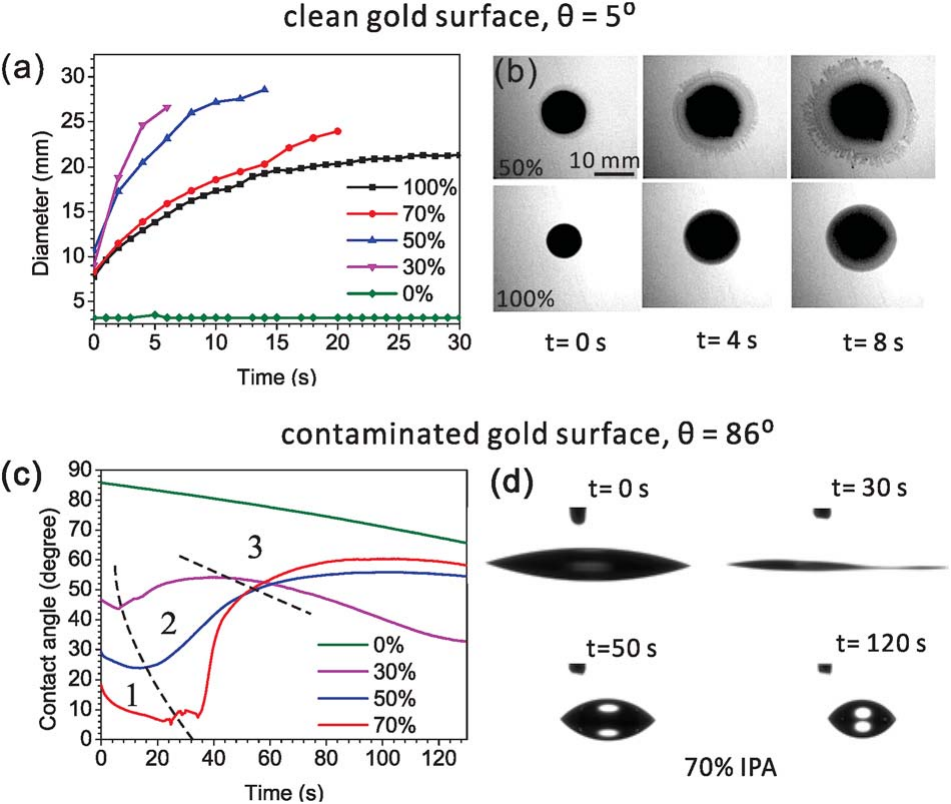
Evolution of IPA–water mixture drops with different IPA concentrations on a clean gold surface: (a) contact diameters and (b) top view images taken at different times for 50% and 100% IPA concentrations. The evolution of drops on a contaminated gold surface: (c) contact angles, indicating the three typical evolution stages divided by black dash lines: (1) dynamic spreading, (2) fast receding, and (3) slow receding; and (d) side view images of 70% IPA drops taken at different stages.

To activate the Marangoni effect, we use a mixture with two components: IPA and water. The influence of the alcoholic concentration on wetting was first studied on a planar, clean gold surface. IPA–water mixtures spread very well on such surfaces (*θ* = 5°) and thus the formed layers are usually too thin to be observed from side view imaging. Therefore, we looked at top images of the coverage after dispensing the mixture drops (5 μl) on the gold surfaces. Rhodamine B was dissolved in the drops to facilitate visualization. The evolutions of drop sizes for different IPA concentrations are shown in Fig. 2a. During a natural evaporation process at room temperature and in a 35% humidity environment, the mixture drops spread much faster and more extensively compared with the pure water and pure IPA drops. When the drop has a lower concentration of IPA, it spreads faster and larger in a fixed timespan (*e.g.*, 4 s), indicating generation of a stronger internal Marangoni flow. The top view images shown in Fig. 2b were taken of 50% and 100% IPA drops at different times after dispensing. At the edge of the 50% mixture drop, a thinner spreading film driven by a Marangoni stress can be clearly seen. During the dynamic spreading process, instabilities are developed at the wetting front, and the smooth edge of the liquid drop gradually becomes a fingering structure. We do not attempt to estimate the diameters of the dendritic drops, so only the initial spreading stage of the drops are compared in Fig. 2a. However, for wetting nanostructures, we prefer to make the drop spread smoothly across the sensing hot spot regions (*e.g.* nanopores), before it becomes dendritic. This can be controlled by both the positioning of the drop and the timespan before implementing the next step in the experiment.

The wetting behaviors for different IPA concentrations have also been compared on contaminated gold surfaces but from side view imaging. The contaminated surface defined here is a surface which is initially cleaned by oxygen plasma and then becomes hydrophobic (*θ* = 86°) during half an hour of exposure to air. Fig. 2c shows the evolutions of contact angles of drops during the first two minutes of evaporation. As shown in Fig. 2c, the pure water drop is found to be pinned with a slow decrease in contact angles, caused by evaporation. This contact line pinning indicates that gold surface has a high contact angle hysteresis and thus a large resistance to contact line motion. By increasing the alcohol concentration from 0% to 70%, the surface tension of IPA–water mixture is monotonically decreased from 0.072 to 0.024 N m^–1^,^39^ and the initial contact angles are also lowered from 86° to 17° as depicted by Young's equation. In the evolution of an IPA–water mixture drop, we easily observed three stages (shown in Fig. 2c): (1) dynamic spreading, (2) fast receding, and (3) slow receding. The duration time of each stage varies for different concentrations. The first dramatic spreading stage corresponds to the quick evaporation of the IPA, which is more volatile compared to water. Near the three-phase contact line, the diverging evaporation rate results in a local depletion of IPA and this drives a Marangoni flow from the central bulk to the contact line. This dynamic wetting leads to a much better liquid coverage even on contaminated gold surfaces (Fig. 2d, 0 and 30 s, 70% IPA solution). Sequentially, after the alcohol concentration in the remaining drop is dramatically reduced in the first stage, the surface tension of the whole drop is increased. As a result, in the second fast receding stage there is a spontaneous dewetting with an increase in contact angle and a sharp decrease in the liquid–solid contact diameter, as can be seen from the side view images (Fig. 2d, 30 and 50 s). After the dewetting stage, the drop reaches the maximum contact angle. The third stage corresponds to the evaporation of water, resulting with a slowly reduced diameter of the drop (Fig. 2d, 50 and 120 s). Combining the results from the clean gold surface, the drop with a lower alcoholic concentration can generate a stronger flow for depinning, but the timespan of the spreading stage is shorter, which may become a problem in practical wetting applications.

### Nanopore wetting evaluation by SERS

To evaluate the wetting of asymmetric nanogaps, we first looked at the SERS spectra taken from long nanopores, with a sub-10 nm gap and a length of around 1 μm (shown in Fig. S1a†). We selected aminothiophenol (4-ATP) as the reporter for SERS in the analyte solution. Through its Au–S bond, 4-ATP can strongly bind onto the gold surface and cannot be removed by rinsing. Once the hot spot region of a nanopore is fully wetted, 4-ATP can enter into and be adsorbed on it. Thus, we can observe strong SERS signals and qualitatively evaluate the performance of wetting.^23^

**Fig. 3:**
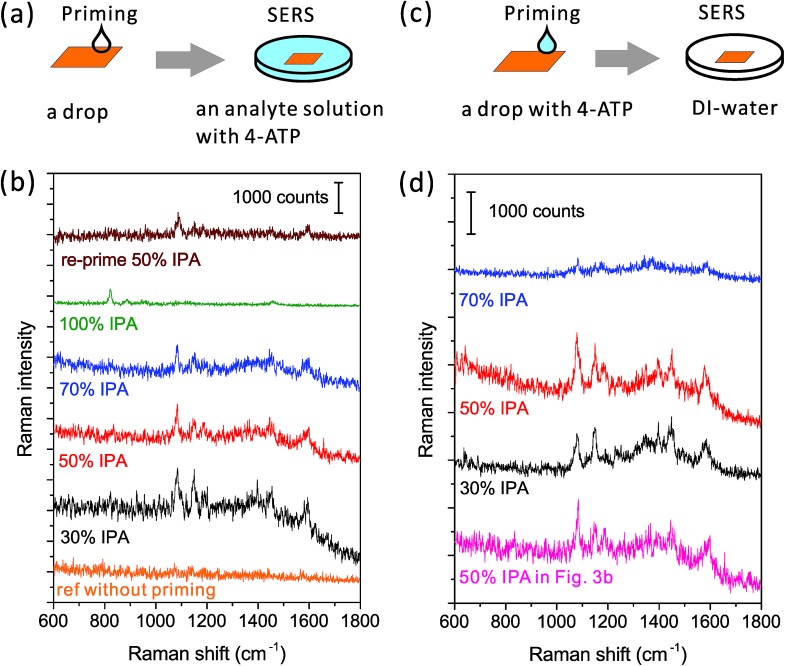
Wetting performance of the priming drop with different alcoholic concentrations. (a and c) Schematic drawings of the experiment process: first prime the nanopore by a drop and then evaluate the wettability of nanopores by SERS. (b and d) SERS spectra from nanopores primed by drops with different IPA concentrations. The difference between the left and right columns is where the Raman analyte contains: in the left, only the analyte solution contains 4-ATP; while in the right, only the priming drop contains 4-ATP. A reference spectrum was taken from the nanopore without priming. The excitation power of the 785 nm laser was ∼2 mW, and the integration time was 1 s. The spectra are off-set.

We initially tried the common wetting method of cleaning the surface. A freshly cleaned nanopore chip was immediately immersed into an aqueous solution with 4-ATP for SERS. However, only a flat background spectrum (shown in Fig. 3b, reference spectrum without priming) was recorded. This clearly indicates that the nanopore has not been fully wetted yet. Most likely, the contact line was pinned outside the hot spot region. As an alternative, solvents with lower surface tensions were then used. According to eqn (1), a solvent like ethanol, acetone or IPA can wet a clean surface better than water. We dissolved 4-ATP in these solvents and used them as the analyte solutions in the SERS evaluation. However, no SERS signals of 4-ATP can be observed, no matter which solvents were used. The capillary effect alone cannot resolve the bubble trapping issues inside nanopores.

However, if we use the priming method based on the Marangoni effect, the nanopore can be fully wetted. A detailed method is shown in Fig. 3a: we place an IPA–water drop near the nanopore, let the drop creep across the nanopore, and then immerse the chip into the analyte solution for SERS. As mentioned above, the strength of the Marangoni flow relies on the alcoholic concentration. Here, we have investigated four IPA concentrations of 30, 50, 70 and 100% in the priming drops. The same nanopore was reused in the study. As shown in Fig. 3b, we can observe very clear SERS spectra of 4-ATP from nanopores primed by all of the mixture drops with different IPA concentrations. These Raman bands at 1075, 1144, 1395, 1441 and 1590 cm^–1^ are attributed to different vibrational modes of 4-ATP.^40^ The intensity of the same Raman bands (*e.g.* 1075 cm^–1^) is similar when the 30 and 50% IPA drops are used, but slightly lower with the use of a 70% IPA drop. It should be noticed that here we only dissolved 4-ATP in the analyte solution for SERS. The weak dependence of the SERS signals on the IPA concentration indicates that the amounts of 4-ATP inside nanopores are similar and the priming performance by different concentrations of IPA is approximately same. We further used a pure IPA drop in the priming and a pure IPA analyte solution with 4-ATP for SERS. As expected, the generated flow inside a pure drop was too weak to move the contact line and only the Raman bands (*e.g.*, 823 cm^–1^) of IPA (Fig. 3b, the green spectrum) can be observed. An interesting re-priming experiment was implemented next. We dried this non-wetted nanopore with a N_2_ gun and exposed it to air for half an hour to make its surface hydrophobic. We then re-primed it by using a 50% IPA drop, and again, we observed a strong SERS signal of 4-ATP (Fig. 3b, the wine colored spectrum). To date, we have applied this priming method to hundreds of nanopores, and the obtained SERS signals were clear and repeatable for each of them.

Although visualizing the dynamic wetting process inside the nanopore remains challenging, we are still curious about using SERS to resolve the influence of the strength of the Marangoni flow on wetting nanopores. To investigate this influence, we need to implement another experiment (Fig. 3c and d). Here, as shown in Fig. 3c, we only added 4-ATP in the priming drops, washed the nanopore chips immediately after priming, and transferred them into analyte-free DI-water for SERS. The same nanopore was reused in this experiment. Since there was no incubation process like the previous experiment, the adsorption of 4-ATP during the short priming interval (2–3 s) was much more related to the dynamic depinning by the Marangoni flow. We have investigated three concentrations of the mixture using drops of 30, 50 and 70% IPA. The resultant SERS spectra are shown in Fig. 3d. We find that the drop with 70% IPA clearly performs significantly worse (∼4× weaker SERS intensity) than the other two drops. In such a short priming (incubation) time, 4-ATP may not fully cover the hot spot region, due to a slower Marangoni flow generated by 70 % IPA drop. On the other hand, the difference between samples primed by 30% and 50% IPA drops is limited. This is consistent with the result from the other priming process (left column of Fig. 3). For selecting a concentration for full wetting, we usually prefer to use the 50%, as it ensures both a good wetting performance and a pretty long dynamic spreading time (tens of seconds) for the leisurely transfer of samples to other solutions. It should also be mentioned that other solvents such as ethanol can be used as alternatives to IPA, if necessary. The priming process also works with use of a 50% ethanol drop (data is not shown here), but may need further optimization of the ratio as well.

### Other wetting methods

We have also considered other wetting strategies such as using pressure, degassing, heating, and electrokinetic wetting. Using pressure to remove the bubbles works for large (>100 nm) nanochannels but is difficult for a sub-10 nm channel, as the capillary pressure (∼*γ*/*d*) can be in the order of 10 atm.^41^,^42^ Degassing the solution in order to decrease the gas solubility and thus remove trapped bubbles was also tested in a low pressure chamber at room temperature, but no SERS signals of analytes were detected after degassing the solutions for half an hour. In our previous experiments,^28^ we found that by applying a voltage across the nanopore membrane, charged molecules like DNA bases or electrolytes can translocate through the gap. This indicates that the energy barrier for spontaneous wetting can be overcome by an electrokinetic effect. However, this method can cause problems of gold corrosion if halogen-based electrolytes and high voltages are used. Another interesting test was heating the chip in hot water (∼80 °C). The heating method was reported to be useful in nanoparticles-based SERS on detecting DNA bases.^43^ In our study, it could also prime nanopores, if the nanopore was only coated by gold on one side. The inhomogeneous boundary of silicon and gold near the nanopore may temporarily form a temperature gradient, which can also cause Marangoni stress.^44^ This pretreatment enabled the nanopore to detect 4-ATP (spectrum is shown in Fig. S2†). However, if both sides of the nanopore are coated by gold, the heating method will not work. The homogeneous boundary cannot generate a temperature gradient or a surface tension gradient. On the other hand, the different performance of the heating method also indirectly proves that using the Marangoni effect is an appropriate way for wetting nanostructures. The disadvantages of using heat are the unsatisfying reproducibility, the long treatment time and the limited universality, all of which prevent it from being an efficient priming method. A summary of all the evaluated methods is listed in Table 1.

**Table 1:** Evaluation of different methods for wetting nanopores

Mechanism	Method	Performance
Surface tension	Immersing into lower surface tension solutions like acetone, IPA, ethanol or a mixture	Failed
Capillary force	A pure drop of acetone, IPA, ethanol or water	Failed
Pressure	Pumping	Failed due to mechanical damage
Degassing	Vacuuming	Failed
Electrokinetic effect	Electrophoresis or electroosmosis^10^,^28^	Successful, but with a risk of corrosion
Marangoni effect	Heating	Successful at heterogeneous surface
Marangoni effect	A mixture drop	Successful

### Universal application for full wetting

**Fig. 4:**
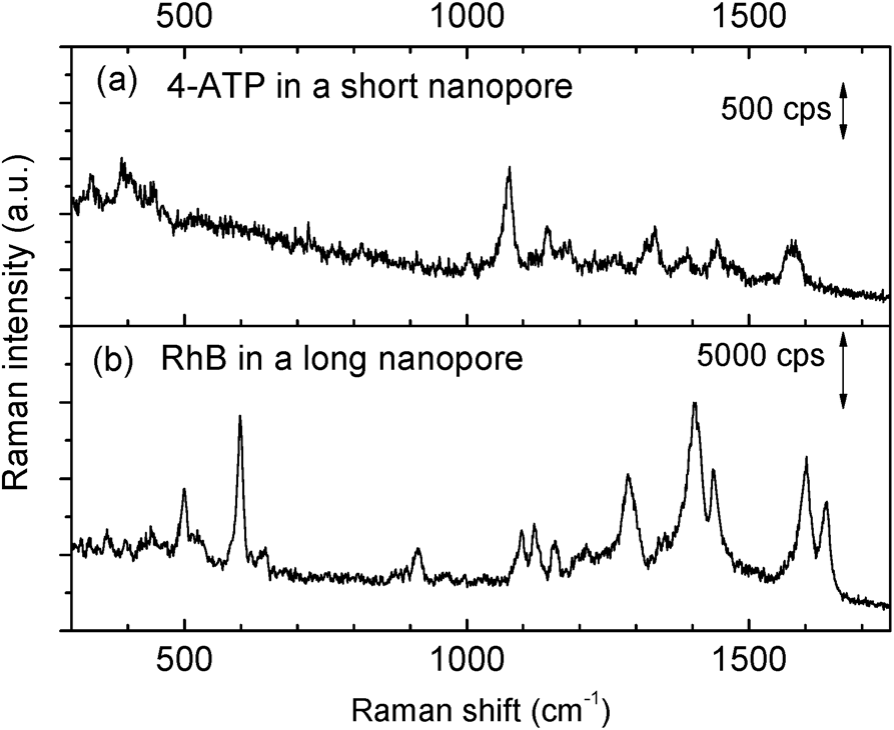
Measured SERS spectra after the priming pretreatment of the structures. (a) SERS spectrum of a 4-ATP SAM taken from the Fabry–Pérot (short) nanopore (13 × 119 nm^2^), power was ∼2.5 mW, and the integration time was 0.5 s. (b) SERS spectrum of rhodamine B (10^–5^ M) taken from the 1 μm long nanopore (∼10 × 1000 nm^2^), and the power was ∼10 mW, and the integration time was 0.1 s.

The priming method with an IPA–water mixture was also proven to work well even for smaller nanopores that have drastically smaller sizes. By shortening the length to match half of the SPP wavelength inside the nanopore, we can harness the first order of the Fabry–Pérot resonance mode for improving nanopore resonant properties.^27^ This strong resonance is extremely useful for fluidic applications.^28^,^45^ However, the small geometrical size (Fig. S1b†), almost an order of magnitude smaller than the longer nanopore we mentioned in Fig. 3, makes bubble trapping a more serious problem.^46^ The electrokinetic effect only works after an application of 2–3 hours rather than several minutes,^28^ which also greatly increases the risk of corrosion of the gold layers. In contrast, the priming method with an IPA–water mixture drop is simple to implement and is also shown to be effective. As shown in Fig. 4a, a clear Raman spectrum of 4-ATP is obtained from a short Fabry–Pérot nanopore with a size of 13 × 119 nm^2^, after the nanopore is primed by a 50% IPA drop. Without the priming step, the obtained spectrum was like the reference spectrum in Fig. 3.

To further study the universality of the method, other types of non-bonded analytes, *e.g.*, rhodamine B (RhB), were also tested. After applying a 50% IPA drop as described before, the chip was immersed into an RhB solution for SERS measurements. The obtained spectrum is shown in Fig. 4b. Raman bands at 603 and 1639 cm^–1^ are aromatic bending modes, 919, 1099, 1125, 1407, 1443, and 1603 cm^–1^ bands are C–H stretching modes, and the 1287 cm^–1^ band is the C–H in the plane bending mode.^47^ Since RhB is a non-bonding analyte, we observed temporal fluctuations of its SERS signal during the measurement. This also confirmed that the nanopore was completely wetted and the molecules could randomly diffuse into and out of the hot spot region following Brownian motion. Again, no SERS spectra could be obtained from RhB without priming.

**Fig. 5:**
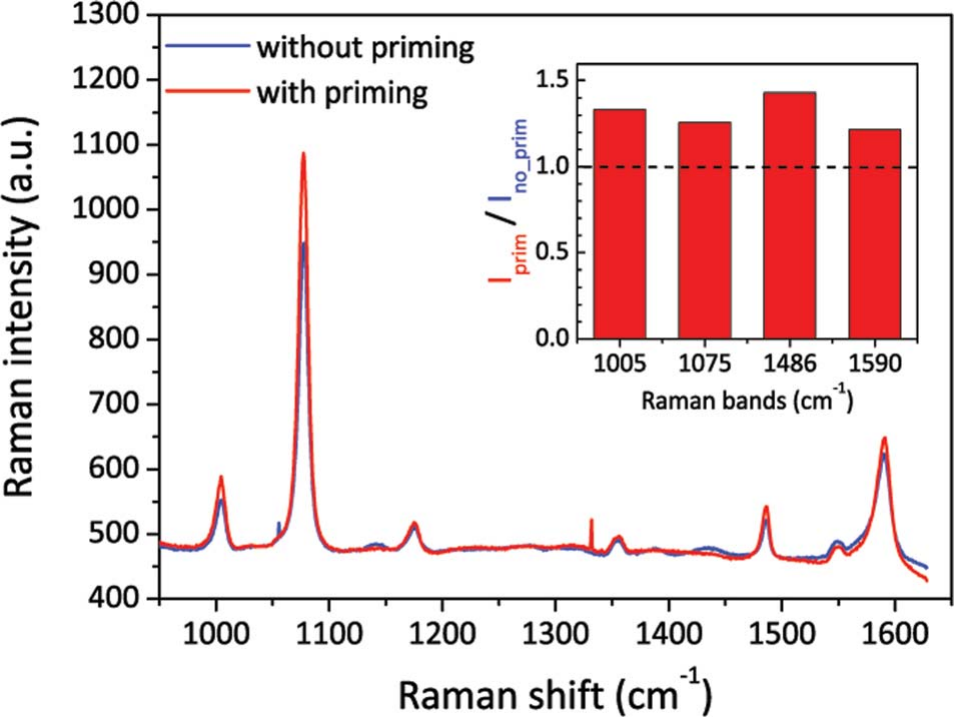
Comparison of SERS spectra measured with Klarite SERS substrates with and without the priming step. The blue and red spectra are the average of spectra taken from 200 same locations from a same Klarite chip. The inserted image shows the intensity ratios of different characteristic Raman bands of 4-ATP, before and after priming. The power was ∼1 mW, and the integration time was 1 s.

We then also applied the priming method to the Klarite SERS substrate,^48^ which is one of the most well-known commercial products. This kind of structure has inverted pyramid arrays in a Si substrate, etched by anisotropic KOH wet etching and coated with a gold layer. Its resonance mainly depends on the depth and pitch of the pyramids. Different to our nanopore-cavity structures, the Klarite substrate has four hot spot regions located at the top edges and one at the bottom vertex.^48^,^49^ These hot spots have similar local optical intensities, but all are weaker than that inside our nanopore-cavities. To figure out the wetting situation of the bottom vertex of Klarite substrates, we introduced our priming method. We used 4-ATP as the analyte, and then linearly scanned the sample over 200 spots with a step size of 1 μm. About 200 SERS spectra were taken by using a low magnification and a low NA objective lens to cover the whole cavity structure. By comparing the average spectrum of these spectra, we can reduce the influence of distinction (defects) of cavities on the Klarite substrates. In Fig. 5, we can clearly see a stronger SERS signals after priming. The integrated intensities of the Raman bands of 4-ATP increased by 20–40%, depending on the different vibration modes. This indicates our priming method can further improve the wettability of such cavities. It is highly possible that the Marangoni effect improves the wetting of the hot spot at the bottom and thus improves the SERS intensity.

## Conclusions

We developed an efficient surface priming method to improve wetting in complicated 3D nanostructures such as the sub-10 nm nanopore as well as the commercial Klarite SERS substrates. The mechanism relies on the solutal Marangoni effect generated by the evaporation of a two-component drop. With the assistance of contact angle and diameter measurements on planar gold surfaces and the SERS evaluation on nanopores, we suggest the use of 50% IPA–water drops for wetting in practice. Due to the diffraction limit, it is difficult to use the conventional optical/fluorescence imaging methods to study local wetting status.^50^ An ultrafast dynamic TEM may provide both high spatial and temporal resolutions to monitor the movement of the contact line, if the confliction of the aqueous sample and the vacuum environment of TEM can be solved.^51^ However, here, we benefit from the intrinsic property of highly localized SERS and the ability to have a nanoscale insight into the wetting status of nanopores. In our SERS evaluations, strong SERS signals, from both surface bonded analytes like 4-ATP and non-bonded analytes like RhB, were only obtained in fully wetted (primed) nanopores. Furthermore, we can also gain a 20–40% increase in the intensity of SERS on Klarite substrates. Compared to other reported pre-treatments for wetting^6^,^28^ or vacuum depositions,^23^ the discussed priming method is much simpler and more universally able to bring analytes into local hot spots of metallic nanostructures. We believe that the two-component drop priming method can be of great interest for emergent applications of plasmonics in fluidics.

## Experimental

Nanopores were fabricated by the standard micromachining process based on e-beam lithography and KOH wet etching. The Klarite chips were ordered from Renishaw Diagnostics. The contact angles were measured using a Dataphysics OCAH 230 system, and the contact diameters were taken by a digital camera, Canon D650. Raman spectra were taken from confocal microscope Raman setups from either Raman α300 (Witec) or LabRAM HR (Horiba Scientific, Ltd), equipped with 785 nm lasers. More experimental details are provided in the ESI.†

## Supplementary Material

Supplementary information
