# Supplementary material for: Full wetting of plasmonic nanopores through two-component droplets
Source: Chem Sci. 2015 Aug 4;6(11):6564–71. doi: 10.1039/c5sc02338f (PMC6054064; doi:10.1039/c5sc02338f)
Supplement: Supplementary file 1 [file SC-006-C5SC02338F-s001.pdf]

Electronic Supplementary Material (ESI) for Chemical Science.
This journal is © The Royal Society of Chemistry 2015

Supplementary information

Full wetting of plasmonic nanopores through two-component droplets

Chang Chen,[a, b,*] XiuMei Xu,[a], Yi Li,[a,c] Hilde Jans,[a] Pieter Neutens,[a, b] Sarp Kerman,[a, b] Guy Vereecke,[a] Frank Holsteyns,[a] Guido Maes,[d] Liesbet Lagae,[a, b] Tim Stakenborg,[a] Pol Van Dorpe,[a, b]

[a]imec, Kapeldreef 75, Leuven 3001, Belgium.

[b]Department of Physics and Astronomy, KU Leuven, Celenstijnenlaan 200D, Leuven 3001, Belgium.

[c]ESAT, Katholieke Universiteit Leuven, Kasteelpark Arenberg 10, Leuven 3001, Belgium.

[d]Department of Chemistry, KU Leuven, Celenstijnenlaan 200F, Leuven 3001, Belgium.

* Email: chang.chen@imec.be; Tel: +3216287794.

EXPERIMENTAL METHOD

Fabrication. The nanopore-cavity chips were fabricated by the standard micromachining process reported before.[1] In brief, it was based on the e-beam/UV lithography, the KOH anisotropic wet etching and metal sputtering deposition. The cavity was etched inside the top silicon layer (700nm) of a silicon-on-isolator chip. After depositing a 100~120 nm Au layer with a 10 nm Ti layer as the adhesion layer on front side of the chip, the resultant size of the nanopore can be adjusted at 5~10 nm in the width and at ~1 µm in the length. If necessary, we can also deposit the metal layers on both sides of the nanopore chip, e.g., the double-side coated gold nanopores discussed in the heating effect (Fig. S3). The double-side coated gold nanopores perform similar optical properties with the single-side coated ones. As shown in Fig. S1a, the gratings near the cavity have a pitch at 316 nm and the width of each groove at 100 nm. Another kind of nanopore was also studied in this work. So-called Fabry-Pérot nanopore with a much shorter length was fabricated by the same processes but with a smaller size at 13 x 119 nm^2. The depth of the cavity is same. A typical structure is shown in Fig. S1b.

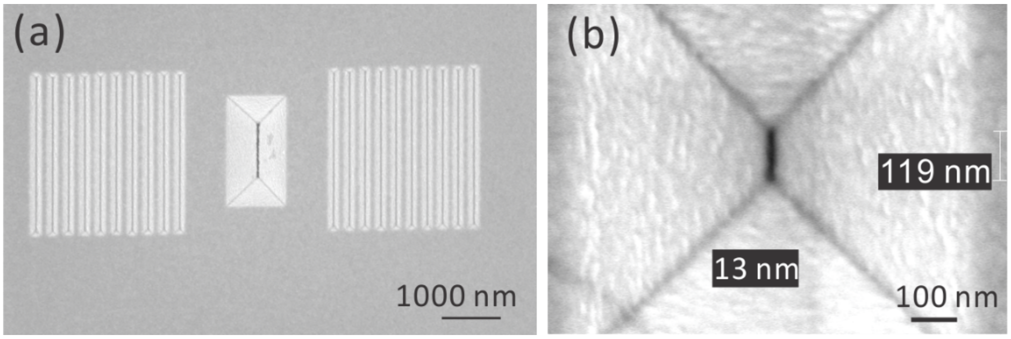

Figure S1. SEM images of (a) the long nanopore with the size at ~10 x 1000 nm^2 and (b) the short Fabry-Pérot nanopore with the size at ~13 x 119 nm^2.

Contact angle and diameter measurement. A 2-inch silicon wafer with a fresh deposited 100 nm Au layer with a 10 nm Ti adhesion layer, was used in the contact angle and diameter measurements. We prepared both clean and contaminated surfaces in the study. The clean surface was prepared by 5 min O_2 plasma treatment just before the measurements. While the contaminated surface was obtained by exposing a clean surface in air for half an hour. The different IPA-water solutions were 0 %, 30 %, 50 %, and 70 %, in volume concentration of IPA. Static contact angles on the contaminated surface were taken from a Dataphysics OCAH 230 system. Sessile drops of 2 µL were placed on the samples through an electronic syringe unit. The contact angles and the normalized contact diameters of drops shown in the time sequence were auto-measured by the software of the equipment, with a manually optimized baseline. As it is difficult to measure the contact angle of drops on the clean surface, we alternatively measure the contact diameters during the evolution of drops. Such measurements were implemented by a camera (Canon D650) fixed above the samples. Rhodamine B was dissolved in the solution to improve the imaging contrast. Drops of 5 µL were placed on samples through a pipette.

No priming process. We cleaned all nanopore chips by 5 min O_2 plasma on both sides before SERS measurements in analyte solutions. 4-ATP (saturated in water or 10 mM in solvents) was dissolved as the Raman reporter in analyte solutions made by different pure solvents. The investigated solvents were IPA, acetone, ethanol, and water. After cleaning, we immediately immersed a whole chip into an analyte solution or placed drops of the analyte solution on one side of the chip, either the front or the back side to evaluate the capillary effect for wetting. However, no matter which solvent we used, we only obtained flat background spectra, instead of clear SERS signals. A typical one without priming is shown in Fig. 3b, the reference spectrum.

Priming process. All chips were cleaned by 5 min O_2 plasma on both sides, and were immediately treated by our priming method. Usually, we used a priming drop of 5 µl. As shown in Fig. S2, during the priming step, the location of placing the drop is not so critical for wetting a nanopore in an open channel. As far as there is a pathway to ensure the evaporation of the mixture drop, both ways of placing the drop near the edge of the chip and directly above the nanopore cavity work for full wetting. However, to wet a nanopore in a closed channel (e.g. Klarite substrate-like cavity with a solid substrate), it is important to place the mixture drop near the edge of the chip. The drop needs to creep into the nanopore cavity for wetting. Usually, we placed the drop as close as possible (3 ~ 5 mm) to the nanopore, on the front side of the chip. To ensure the contact line movessmoothly cross the hot spot regions, we must control the

timespan (e.g. ~ 10 s) for transferring the chip from priming into another aqueous medium. The transfer should be done after the contact line (the edge of the drop) moves cross the nanopores, but before the drop starts being dendritic or receding. Once the chip was in the medium, it was safe for keeping in wet state. In this work, we studied the influence of the alcoholic concentration of the priming drops on the wettability of nanopores. Two kinds of priming drops were prepared. The first kind was analyte free mixtures with IPA volume concentrations of 30 %, 50 % or 70 %, (the left part in Fig. 3). After washing in DI-water three times, we incubated the samples in the analyte solution with 4-ATP for 10 minutes before SERS. A same nanopore chip was reused in all concentration experiments after thorough cleaning (checked by SERS). The second kind had same concentration variations, but with pre-dissolved 4-ATP (the right part in Fig. 3). The nanopore was immediately washed three times after priming, and SERS was implemented in analyte-free DI-water.

We also evaluated the wetting performance of a pure alcohol like IPA. We used a pure IPA drop without 4-ATP and an IPA-based analyte solution with 4-ATP (~ 10 mM). The nanopore was primed by a drop of IPA in the same way. Then we directly immersed the chip into the analyte solution. Since we used IPA as the only solvent in the analyte solution, it was not necessary to wash the chip.

We further studied the feasibility of re-priming. We used the chip tested in the pure IPA priming, dried the chip by N_2 gun, without O_2 plasma cleaning. Since this sample was already used and exposed in air for around half an hour, the surface became hydrophobic. We then placed a drop of 50% IPA to prime the nanopore. After washing 3 times by DI-water, we evaluated it in the analyte solution with 4-ATP by SERS.

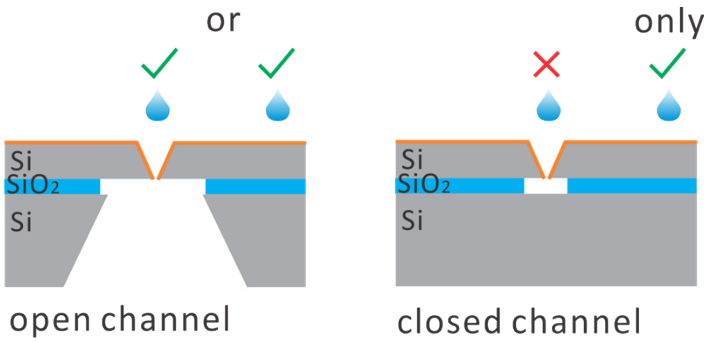

Figure S2. The schematic drawing of the priming step. When the nanopore is in a freestanding membrane, it is not critical of the location for placing the drop; when the nanopore is on a solid substrate, it is better to place the drop near the edge of the chip.

SERS measurement. Witec α300 Raman setup, a confocal microscope equipped with a 785 nm laser and a spectrometer with an EMCCD (DU970N-FI, Andor), was used for the measurement. The grating was 600g/mm, supporting a spectral resolution at ~ 1.2 cm^{-1}.

The feasibility of using a two-component priming drop was mainly proved by investigating SERS of the long nanopores (10 x 1000 nm^2). After preparing the nanopores in the analyte solution, the SERS spectra were taken at ~ 2 mW excitation and 1 s integration time by using a water immersion lens (60x, NA= 0.9). The spectra shown in Fig. 3 are the raw data without any treatments. The Fabry-Pérot nanopore (13 x 119 nm^2) with gratings was used in the study of the universality of the priming method for structures. We first primed it by a clean priming drop (50% IPA, 4-ATP free), and then washed it three times by DI-water. After incubated for an hour in the

analyte solution with 4-ATP, we took the spectrum at ~ 2.5 mW excitation and 0.5 s integration time.

Rhodamine B (10^{-5} M, in water) was used in the study of the universality of the priming method for specimens. We primed the chip by a clean drop and washed in DI-water three times. The SERS spectra were taken at ~ 10 mW excitation and 0.1 s integration time.

Other tested priming methods for full wetting.

1. **Degassing.** We have studied two ways. In the first way, an analyte solution with 4-ATP was degassed in a low pressure chamber for half an hour. Then a freshly-cleaned nanopore chip was immediately immersed into it for at least half an hour incubation. In the second way, we immersed a clean chip into the analyte solution and then placed in the vacuum chamber for degassing for half an hour. The chips were directly checked in analyte solutions, but no SERS signals could be detected from these chips.
2. **Heating.** A 600 ml beaker filled with 300 ml pure water was heated to ~ 80 °C by a hotplate. Two kinds of chips, one with a single-side gold coating, and the other with a double-side gold coating, were studied. They were both immersed into the hot water for 10~20 minutes. Then the pretreated chips were immersed into the 4-ATP solution for incubation of an hour before SERS. As shown in Fig. S3, it can record a clear SERS spectrum (black) from the single-side (SS) coated gold nanopore. However, the one taken from the double-side (DS) coated sample was only background noise, no 4-ATP SERS can be detected (red). These Raman spectra were recorded from LabRAM HR (Horiba Scientific, Ltd), using 1 mW power and a water immersion objective lens (60 x, NA = 0.9) and the integration time was 1 s for each sample. A 600 grooves/mm grating was used to obtain a spectral resolution of 1.5 cm^{-1}.

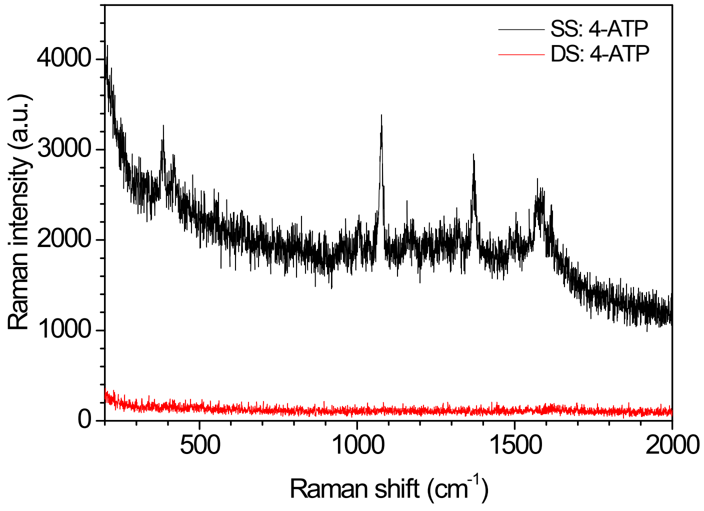

Figure S3. SERS spectra taken from single-side (SS) coated nanopores (black) and a double-side (DS) coated nanopore (red) in 4-ATP solutions.

Klarite substrates measurement

The Klarite chips were used to study universal application of priming in different structures. The samples were ordered from Renishaw Diagnostics (Fig. S4), with a pyramid size of ~ 1.6 µm and the pitch at ~ 2 µm. The measured resonances in air are ~ 600, 800, 1100 nm, while in water

they are red-shifting. Each chip was cleaned by O_2 plasma before use. We first prepared a reference experiment before priming. The clean chip was directly immersed into 4-ATP (~ 10 mM) ethanol solution for an incubation of 3 hours. In the priming experiment, we reuse the same chip after the O_2 plasma cleaning, and then placed a 50 % IPA drop without 4-ATP near the edge of the chip for priming. Later we took an incubation of 3 hours. In both experiments, we had to rinse the chip three time by ethanol to remove the non-absorbed molecules before taking spectra. The samples were measured in air by using a Horiba LabRAM HR Raman setup with a 785 nm laser at 0.2 mW. A linear scan with 200 spots and a step at 1 μm was implemented at the same locations on the same chip during each experiment. Each spectrum was acquired for 1 s and accumulated once. The grating used was 600 g/mm. To cover more pyramids, a 10 x air objective lens (NA = 0.25) was used to excite structures and collect the Raman scattering. The estimated laser spot was ~4 μm. We compared the averaged spectrum taken from these 200 spots in each experiment, and concluded that the priming step does help for wetting Klarite substrates as well (shown in Fig. 5).

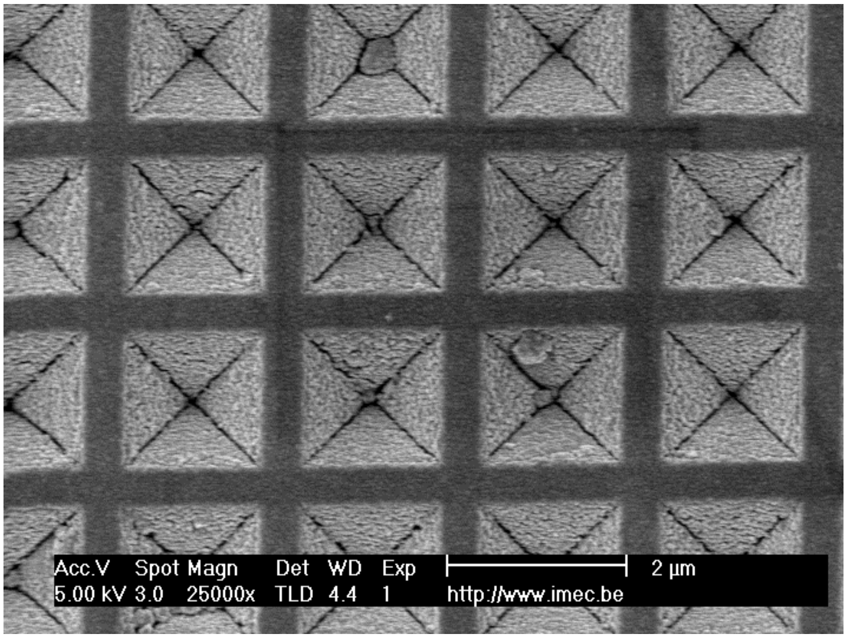

Figure S4. SEM image of a typical Klarite chip.

REFERENCES

1 C. Chen, J. A. Hutchison, P. Van Dorpe, R. Kox, I. De Vlaminck, H. Uji-I, J. Hofkens, L. Lagae, G. Maes and G. Borghs, *Small*, 2009, **5**, 2876–2882.
